# A Public Warning

**Published:** 1921-04-09

**Authors:** 


### A PUBLIC WARNING.

It is not easy to count from memory the number of "cures" which have in recent years been presented to the public in the pages of the daily press. In any case they are legion. Most recently it has been tuberculosis; to-morrow it may be cancer or any other disease of high incidence and mortality rates. But surely it is time to follow the example set by one of the less sensational evening papers in sounding a note of public warning. In dealing with national medical problems it is manifestly absurd to buoy up the individual sufferer with false hopes. Still more detrimental to public welfare is the turning of unconfirmed medical statements to the purposes of political propaganda. Writing essentially from the professional and practical standpoints, one would put no limit to the possible advances of curative medicine. New "cures" and new methods will undoubtedly materialise. But the public must be warned against accepting the promise as the accomplished fact.

With regard to the widely boomed Spahlinger treatment, Sir Robert Philip recently reported some of the communications he had received from the Paris Academy of Medicine. In this case the authoritative statement was that, after receiving serious consideration, the Academy decided the claims of the Spahlinger treatment to have no substantial basis. Again, although in his own paper on vaccination and tuberculosis Dr. Nathan Raw makes his meaning perfectly clear, the press has on occasions come perilously near misrepresenting the whole thing. In a note it is impossible to enter into technical details, but at least it may be said that the author suggests a method of *preventing* tuberculosis in children. There is a world of difference between this project and the discovery of a specific "cure" for tuberculosis in general.

Numberless other examples might be given, and one is prepared to admit that the source of many of these public statements is in itself sound enough. We are fully in favour of widespread publication of discoveries, but editors must insist on both accuracy and moderation from their contributors if their readers are not often to suffer the bitterest disillusionment.

